# Bacterial evolution during human infection: Adapt and live or adapt and die

**DOI:** 10.1371/journal.ppat.1009872

**Published:** 2021-09-09

**Authors:** Matthew J. Culyba, Daria Van Tyne

**Affiliations:** 1 Department of Medicine, Division of Infectious Diseases, University of Pittsburgh School of Medicine, Pittsburgh, Pennsylvania, United States of America; 2 Center for Evolutionary Biology and Medicine, University of Pittsburgh School of Medicine, Pittsburgh, Pennsylvania, United States of America; Carnegie Mellon University, UNITED STATES

## Abstract

Microbes are constantly evolving. Laboratory studies of bacterial evolution increase our understanding of evolutionary dynamics, identify adaptive changes, and answer important questions that impact human health. During bacterial infections in humans, however, the evolutionary parameters acting on infecting populations are likely to be much more complex than those that can be tested in the laboratory. Nonetheless, human infections can be thought of as naturally occurring in vivo bacterial evolution experiments, which can teach us about antibiotic resistance, pathogenesis, and transmission. Here, we review recent advances in the study of within-host bacterial evolution during human infection and discuss practical considerations for conducting such studies. We focus on 2 possible outcomes for de novo adaptive mutations, which we have termed “adapt-and-live” and “adapt-and-die.” In the adapt-and-live scenario, a mutation is long lived, enabling its transmission on to other individuals, or the establishment of chronic infection. In the adapt-and-die scenario, a mutation is rapidly extinguished, either because it carries a substantial fitness cost, it arises within tissues that block transmission to new hosts, it is outcompeted by more fit clones, or the infection resolves. Adapt-and-die mutations can provide rich information about selection pressures in vivo, yet they can easily elude detection because they are short lived, may be more difficult to sample, or could be maladaptive in the long term. Understanding how bacteria adapt under each of these scenarios can reveal new insights about the basic biology of pathogenic microbes and could aid in the design of new translational approaches to combat bacterial infections.

## Introduction

The study of microbial evolution has profound implications for improving public health. Understanding the genetic changes that enable pathogens to adapt and persist in their hosts uncovers fundamental biology and leads to new therapeutic interventions. For example, monitoring antibiotic resistance among circulating bacterial populations enables the rational selection of empiric antibiotic therapy, as well as prioritization of research that aims to develop novel interventions [1]. On an individual level, tracking within-host evolution, or the genetic changes within a microbial population infecting a single patient, also leads to critical insights. One prominent example of this, which occurred prior to the advent of modern DNA sequencing, was the development of multidrug regimens to treat tuberculosis to combat the predictable emergence of phenotypic drug resistance during therapy [2]. Now, with greater accessibility of next-generation DNA sequencing technologies and modern analytical tools, our understanding of the evolutionary dynamics underlying these processes is expanding. Within-host evolution can now be studied at high resolution, and, potentially, in real time [3]. This provides a window into the in vivo evolutionary dynamics that underlie the emergence of antimicrobial resistance, as well as a wider range of traits important for pathogenesis, persistence, and transmission. With minimal modifications, the same analytical principles applied to in vitro microbial evolution experiments can also be employed in in vivo studies. Indeed, clinical infections can be viewed as naturally occurring experiments in microbial evolution.

In this review article, we discuss the similarities and differences between studying bacterial evolution in vivo versus in vitro, as well as the challenges and opportunities presented by studying within-host bacterial evolution during infection in humans. We begin by discussing some of the major differences between studying bacterial evolution in vitro versus in vivo. Then, we present 2 different ways of thinking about adaptive mutations in vivo, which we have termed “adapt-and-live” and “adapt-and-die.” Finally, we offer practical considerations for conducting studies of bacterial evolution in vivo during human infection. Our goal is to persuade researchers and clinicians that it is feasible to study bacterial evolution in vivo, and that much can be learned from conducting such studies.

### Evolutionary dynamics during experimental microbial evolution

In vitro experimental microbial evolution has proven to be a powerful tool to understand the evolutionary dynamics within a bacterial population as it adapts to a new environment [4]. In a typical experiment, a bacterial strain is subjected to serial passaging, where it is exposed to a defined selective pressure, setting the process of evolution in motion. To gain information about the reproducibility of evolution, experiments are conducted with multiple replicates. The bacterial population can be sampled frequently throughout the experiment and, in the present era, subjected to whole genome sequencing (WGS) to track the fates of mutations that rise and fall in frequency through time. Samples of the population can be cryopreserved indefinitely and resuscitated at any time for additional characterization. The classic example of in vitro microbial evolution is the *Escherichia coli* long-term evolution experiment (LTEE), which was initiated by Richard Lenski more than 30 years ago [5], continues to this day, and has now exceeded 75,000 bacterial generations. More commonly, however, in vitro evolution experiments occur on timescales spanning days to weeks. The technique has also been applied to a growing number of different microbes, including yeasts [6], viruses [7], and bacteriophages [8,9]. Importantly, experimental conditions can be adjusted to explore specific phenotypes. For example, plastic beads can be used as a surface for biofilm formation, which enables specific transfer of the biofilm population into fresh media during serial passaging. This method has been used to study pathogens such as *Burkholderia cenocepacia* [10,11] and *Pseudomonas aeruginosa* [12] to uncover biofilm-specific signatures of evolution.

Evolution is extraordinarily dynamic, even when studied in a static, well-controlled environment. In the LTEE, the glucose-limiting growth medium and the passaging procedure remained constant, yet dramatic changes in bacterial biology still occurred. By 20,000 generations, the bacteria could grow 70% faster in culture [13]. After 30,000 generations, one of the 12 replicate populations gained the ability to import citrate from the culture medium and use it for aerobic growth [14]. Six populations acquired defects in DNA repair, elevating their mutation rates by approximately 100-fold [15]. Finally, fitness measurements from the most recent analysis, after 60,000 generations and more than 25 years, showed that the bacteria continued to enhance their ability to grow in the media [16,17]. Thus, even in a stable environment, spontaneous mutations provide an ample supply of genetic diversity to continuously fuel the successive evolution and refinement of new traits. Furthermore, because the *E*. *coli* population chosen for the LTEE is asexual, meaning that the bacteria lack plasmids or bacteriophages that could mediate horizontal gene transfer (HGT), evolution occurs solely by mutation, genetic drift, and natural selection. In the absence of HGT, mutations cannot be shared between different clones. This results in an especially strong linkage disequilibrium of mutations and a high degree of clonal interference. Clonal interference occurs when beneficial mutations that arise in different clones compete against each other, which can cause a beneficial mutation present in one clone to be extinguished from the population as it is outcompeted by a different, more fit clone [18]. In sexual bacterial populations, some mutations can be shared. In this case, the population size and the rate of gene exchange become important factors that determine the adaptive advantage of HGT [19]. Notably, HGT is a pervasive mechanism in the evolution of antibiotic resistance in clinical settings [20].

Experimental conditions greatly influence the timescale of evolution. The identical citrate utilization pathway that evolved after 30,000 generations during the LTEE can be selected for in just 12 to 100 generations if the population is subjected to prolonged selection during nutrient starvation [21]. Similarly, DNA repair defects that elevated the mutation rates in the LTEE can be evolved by chemically mutagenizing an *E*. *coli* population and immediately applying 2 rounds of different selective antibiotics [22]. The conditions of these experiments differ from the LTEE because they enable the bacterial population to survey much greater genetic diversity, while also being subjected to stronger selective pressure. The effect of population size (*N*) on evolution is also a critical parameter. First, it determines the minimal effect size of a mutant allele (i.e., the relative fitness advantage conferred) that natural selection can detect, with small effect sizes requiring larger *N* in order to be selected [23]. Second, due to spontaneous mutations, *N* greatly contributes to the amount of genetic diversity available for selection, with diversity increasing in proportion to *N*. Although stochastic phenomena have a greater influence in small populations and can occasionally lead to big leaps of adaptation across a fitness landscape, the greater sensitivity to selective forces and the more genetic diversity that is present within large populations tends to favor adaptive potential [24]. These basic principles of population genetics are critical considerations for investigating evolutionary phenomena, both in vitro and in vivo.

### Bacterial evolution in vitro versus in vivo

Human infections occur in a complex environment. This in vivo complexity is evident when considering the numerous tissue microenvironments that can be accessed by a bacterial population within a single individual. Bacteria may colonize different anatomical sites without causing disease (e.g., skin surface and gut lumen), and then invade a variety of different tissue compartments (e.g., soft tissue, blood, bone, joints, and lungs) within an individual host. Each tissue type represents a distinct microenvironment, and a unique fitness landscape, for the infecting bacteria. If an infection spreads within an individual, the bacteria are likely to experience population bottlenecks at tissues interfaces. Bottlenecks also occur during transmission between individuals, since relatively few bacteria initiate infection or colonization of new hosts. Due to the small *N* of initial founder populations, the statistical phenomenon of genetic drift will have a strong influence on mutant allele frequencies early during infection. As the population expands, however, spontaneous mutations supply genetic diversity for the production of new traits. Mutant alleles associated with beneficial traits can then rise in frequency due to positive selection from new tissue microenvironments, immune responses, and antibiotic treatments.

Although many infecting populations can be expected to evolve rapidly, studying this process can be difficult due to several practical constraints. First, an infection must either be persistent or chronic to allow for serial sampling of the bacterial population over time. Sampling should also start early enough in the infection so that the population can be assessed before substantial selection has occurred. Second, the sampling method must capture sufficient genetic diversity to be able to detect genetic changes through time. Outside of a dedicated clinical trial, however, sampling is usually limited to clinical specimens collected for diagnosis during the routine care of the patient, which may be limiting. A third challenge is that the parameters that govern the evolutionary dynamics of the infecting population, such as population size, rates and sources of mutation, and the nature of the selective pressures, must be expected to produce detectable mutations within the timescale of the infection. Finally, the infection must be common enough to accrue a sufficient number of independent individuals and infections for study, so that evidence for parallel or convergent evolution can be pursued and more general conclusions about evolutionary signals can be made. Due to these constraints, not all bacterial infections are suited to this type of study.

**Fig 1:**
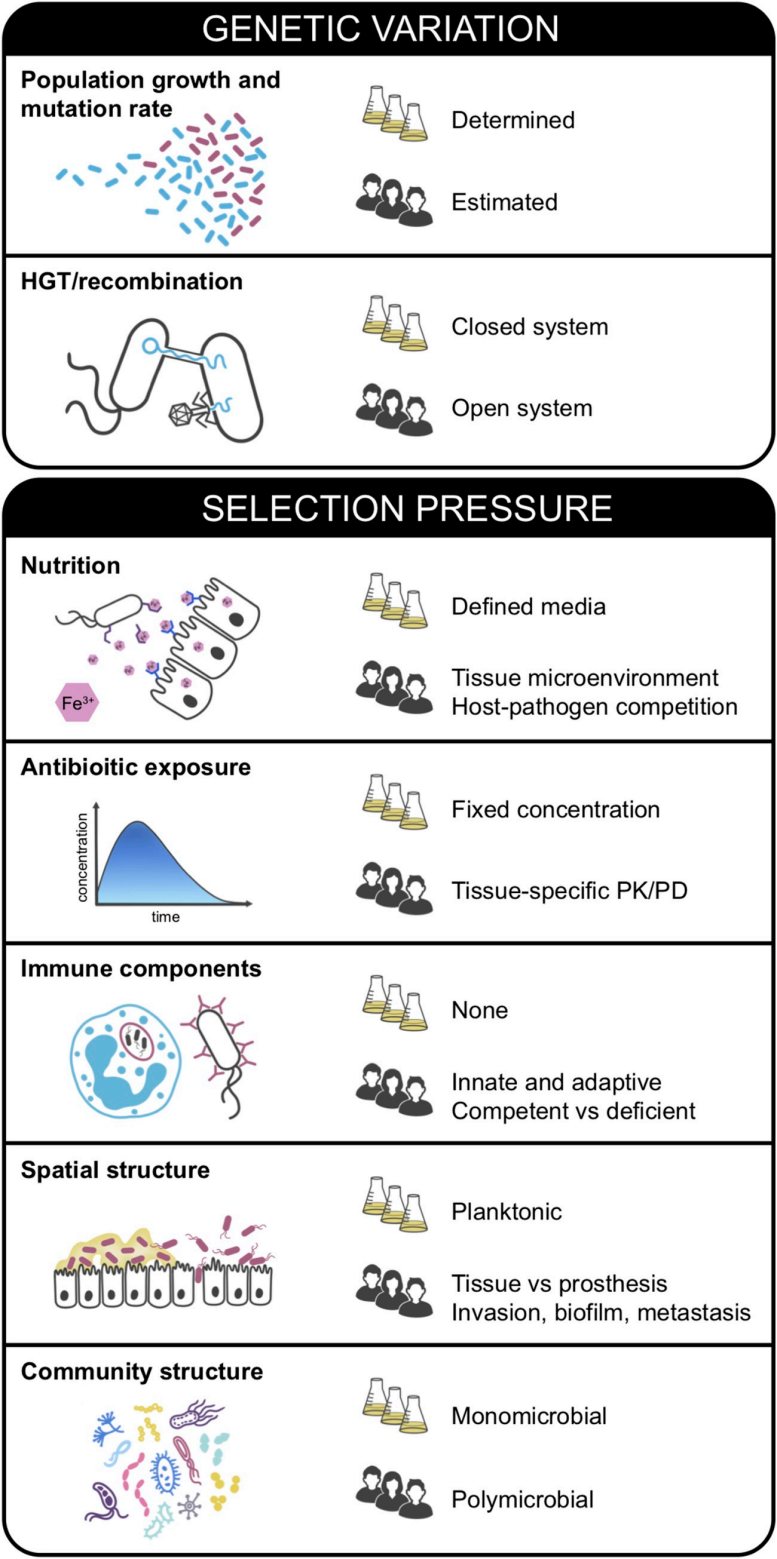
In vitro versus in vivo bacterial evolution. Comparison of parameters that differ between bacterial evolution in vitro (flasks) and in vivo (people). HGT, horizontal gene transfer; PK/PD, pharmacokinetics/pharmacodynamics.

Beyond the practical limitations, the primary differences between in vitro and in vivo systems are that (i) the amount of genetic variation and (ii) the nature of the selective pressure(s) are much more dynamic in vivo (Fig 1). In vitro, bacterial population size, growth rate, mutation rate, and mobile genetic element movement can all be determined. Whereas, in vivo, these parameters can often only be estimated, and they can also be variable between patients. Perhaps the most important difference between in vitro and in vivo settings, however, is the nature of the selective pressures. In vitro, environmental conditions are experimentally defined, such as the concentration of an antibiotic, the specifics of the growth medium, and even the spatial structure of the microbial population. For example, a recent in vitro study of *Acinetobacter baumannii* compared the evolution of antibiotic resistance between cells growing in planktonic cultures with cells growing in biofilms [25]. Due to the ability to precisely define all other experimental variables, this study was able to conclude that the differences observed in the evolution of antibiotic resistance were due to the spatial structures of the bacterial communities. Such control is not possible in vivo, where selection is almost certainly due to a combination of factors (Fig 1). Patient infections occur in a variety of different tissue microenvironments and commonly involve implanted medical devices, such as prosthetic joints, central venous catheters, or cardiac pacemakers. Each site of infection is likely to differ in terms of available nutrients, antibiotic exposures, immune responses, spatial structure, and microbial community structure (Fig 1). Some of these features have been explored by in vitro studies, for example, through the use of synthetic media that more accurately mirrors in vivo nutrient conditions [26], variable antibiotic selection regimens [27], or in vitro studies of polymicrobial interactions [28]. Despite these advances, however, the complexity of many in vivo environments is unlikely to ever be perfectly mirrored in vitro. Nonetheless, we do not view the many unknowns of the in vivo environment described above as a hindrance for discovery. Quite to the contrary, studying bacterial evolution in vivo poises investigators to make novel discoveries. We believe that studying within-host evolution can serve as the starting point for a discovery pipeline that produces important insights into the causal mechanisms underlying bacterial pathogenesis, persistence, and transmission.

### Adapt-and-live versus adapt-and-die mutations

Many in vivo microbial evolution studies are focused on identifying mutations; more specifically, de novo mutations, which confer an adaptive advantage. Because sampling of bacterial populations is nearly always limiting, there is an inherent detection bias toward high-frequency mutations that are long lived. This bias poses a practical barrier to identifying short-lived and low-frequency mutations that are still of great interest. With this in mind, here, we categorize de novo adaptive mutations into 2 groups, using the timescale in which a mutation persists after its inception as the distinguishing feature between groups:

Adapt-and-live mutations are long lived (months to years) and are associated with chronic infections or asymptomatic colonization. They may or may not be transmitted to other people, but the opportunity for transmission is higher given that they persist for longer timescales. Some mutations may even enhance pathogen transmission.
Adapt-and-die mutations are short lived (days to weeks). They arise due to their selective advantage but are then rapidly extinguished. In acute infections, this is often because the infection resolves, or it is at an anatomic site that represents an evolutionary “dead-end” (see below). During chronic infections, the demise of these mutations is primarily due to the evolutionary dynamics of fitness trade-offs, epistatic effects, clonal interference, or stochastic elimination due to a sudden drop in population size. These mutations are much less likely to be transmitted to other hosts, meaning that they arise independently in each newly infected host.

### Adapt-and-live mutations: Evolution of beneficial traits that persist

When bacteria cause infections in humans, they often encounter environments that are quite different from the natural environmental niches to which they are well adapted. For example, bacteria that naturally reside in soil or water can cause opportunistic infections in humans, where they will experience large differences in temperature and available nutrients, as well as the presence of antibiotic and immune selective pressures. If the bacteria are able to adapt to these new conditions, they will be able to divide more rapidly, persist longer before dying, and survive long enough to find a new home within the same or a different host. As researchers, we want to understand how bacteria evolve during human infections. We frequently do this by identifying and characterizing mutations that occur in bacterial populations during the course of infection. Chronic bacterial infections offer the opportunity to study these adaptations on timescales from months to decades. In this section, we focus on 2 primary examples of adapt-and-live mutations, in which genetic adaptations that occur during infection prolong bacterial survival, either through transmission to new hosts or through the establishment of chronic infection.

**Fig 2:**
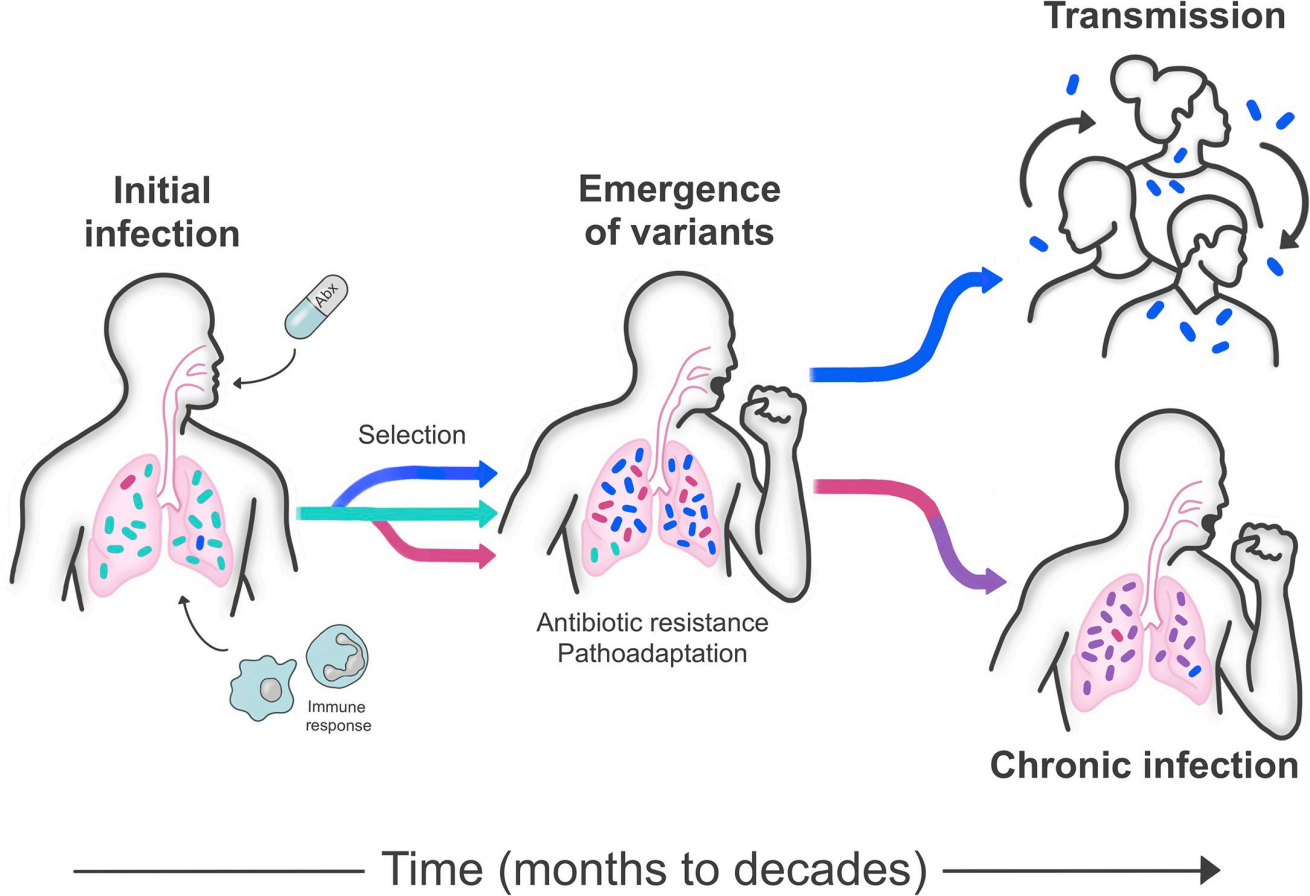
Bacterial evolution during chronic infection. Pulmonary infection is shown as an example. Infection starts with a population of bacteria entering a new environment, such as the lung. Antibiotics, immune responses, and other pressures exert selection on the bacterial population, causing the emergence of bacterial variants possessing antibiotic resistance and other adaptive traits in a process referred to as pathoadaptation. Bacteria from the adapted population can be transmitted to other individuals or can continue to adapt and cause chronic infection, lasting months to decades.

One example of an adapt-and-live scenario is the evolution of antibiotic resistance during active pulmonary infection with *Mycobacterium tuberculosis*. After reactivation of a latent tuberculosis infection, active disease can last for many years; this long timescale, combined with intrinsic tolerance to many antibiotics and poor antibiotic permeability, allows the bacteria to evolve resistance [29]. In contrast to many other bacteria, where antibiotic resistance can be acquired through HGT, *M*. *tuberculosis* evolves resistance de novo through the accumulation of mutations in genes encoding antibiotic targets, regulators of those targets, as well as the up-regulation of efflux pumps [30]. Numerous studies have described the development of antibiotic resistance during *M*. *tuberculosis* infection in humans, first using molecular typing and more recently using WGS [31–33]. One of the more troubling features of antibiotic resistance in *M*. *tuberculosis* is that resistant bacteria can be transmitted from infected to uninfected hosts [34] (Fig 2). While antibiotic resistance mutations frequently carry fitness costs, in *M*. *tuberculosis*, these costs can be mitigated by compensatory mutations [35], which enable the bacteria to propagate through human populations even in the absence of ongoing antibiotic selection.

A second example of an adapt-and-live scenario is chronic pulmonary infections with *P*. *aeruginosa* and *Burkholderia* species, which have been most intensely studied in patients with cystic fibrosis (CF). Similar to *M*. *tuberculosis* infections, patients with CF are often infected for years to decades with a population of bacteria in which genetic and phenotypic diversity arise over time [36–38]. Selection on this population drives a gradual process of pathoadaptation, ultimately resulting in a chronic and antibiotic-resistant infection [39] (Fig 2). WGS offers an opportunity to observe how this process unfolds on both short and long timescales [40,41]. A recent study of over 400 *P*. *aeruginosa* isolates sampled longitudinally from 39 young CF patients over 10 years found that pathoadaptation in the CF lung involves an initial 2- to 3-year period of rapid adaptation, followed by a transition to persistent infection [40]. During the initial period, bacterial growth rate was found to slow and antibiotic resistance increased through accumulation of ciprofloxacin resistance–associated mutations in *gyrA/B* and mutations in the efflux pump repressor *nfxB*. Infections were then able to persist long term via both convergent evolution [42], as well as through the maintenance of a genetically diverse population [43,44]. These populations remodel their regulatory and metabolic networks (largely through mutations in transcriptional regulators), gain the ability to adhere more proficiently (driven by biofilm-increasing mutations and loss of flagella), develop mucoid and/or hypermutator phenotypes, produce fewer extracellular virulence factors, and acquire antibiotic resistance [45,46]. These prior studies have shown that even among different patients and genetically diverse *P*. *aeruginosa* populations, evolution is relatively predictable in this setting.

Many of the above themes are repeated when considering the adaptation of *Burkholderia* to the lungs of CF patients. A landmark 2011 paper described a retrospective study of 112 *Burkholderia dolosa* genomes from 14 individuals with CF that were isolated over 16 years [47]. The authors identified parallel adaptive mutations in antibiotic resistance genes (DNA gyrase subunits *gyrA/B*, ribosomal protein *rpl4*, and others), genes involved in outer membrane synthesis (such as glycosyltransferases *wbaD* and *mtfA*), and the two-component system *fixLJ*, which senses oxygen tension and governs biofilm formation, motility, and persistence within macrophages [48]. A similar study of *Burkholderia pseudomallei* evolution during chronic infection in 7 CF patients identified parallel mutations in genes conferring antibiotic resistance, genes in the type 3 and type 6 secretion systems that were predicted to decrease virulence, and fatty acid biosynthesis genes including *fabF* and *fabG*, which are predicted to impact membrane fluidity and permeability [49]. Genes involved in lipid transport and metabolism were also identified as likely targets of selection in a separate study of long-term evolution of *Burkholderia multivorans* over 20 years in a single CF patient [50]. Over the decades-long timescales documented in several studies, it has also been possible to observe reductive evolution in both *B*. *cenocepacia* and *B*. *pseudomallei* [37,49]. This process of genome “slimming” often happens during long-term adaptation and specialization within a defined host [51]. Finally, a study of 32 *B*. *cenocepacia* isolates from 8 CF patients identified parallel mutations in the RNA polymerase subunit *rpoB*, catalase *katG*, the copper sensor kinase *cusS*, and the methionine-sulfoxide reductase *yedY* [52]. The functional consequences of these mutations, and how they impact bacterial survival during chronic infection in CF patients, remain to be determined.

In all of the above examples, the patients that were studied can be considered as single observations within larger in vivo evolutionary experiments. Combining the results of these replicates across studies reveals several common themes of bacterial evolution in the adapt-and-live scenario. First, evolution of antibiotic resistance is clearly important in the setting of chronic bacterial infections. This is likely because antibiotic treatment imposes strong selective pressure, and resistance-conferring mutations have a large effect size [53,54]. Second, a highly variable suite of mutations occur that impact the ways that the bacteria interact with their host. These include mutations that modulate bacterial virulence, often by down-regulating acute virulence in favor of strategies that promote persistence in the face of innate and adaptive immune pressures [38,55–57]. Third, as was also observed in the in vitro LTEE, hypermutators frequently arise. Rapid evolution of hypermutators in vitro is facilitated by serial exposure to strong and varying selection pressures [22]. This observation likely explains the frequent emergence of hypermutators in the CF lung, where bacterial populations are able to persist in the face of variable antibiotic treatments and a dynamic host immune response [49,58,59]. Emergence of hypermutator strains is clinically concerning, as it portends rapid acquisition of antibiotic resistance [60], and may be a marker of disease progression in CF [61]. While these appear to be some of the common “rules” that govern the evolution of *M*. *tuberculosis*, *P*. *aeruginosa*, and *Burkholderia* during chronic infection, it is important to note that genotypic and phenotypic heterogeneity are frequently found across studies [44,62]. This highlights the dynamic nature of bacterial evolution and points to a need for deep sampling of many patients in order to gain a more complete understanding of bacterial adaptation in the adapt-and-live scenario.

### Adapt-and-die mutations: Evolution of beneficial traits that do not persist

In contrast to adapt-and-live mutations, adapt-and-die mutations are ultimately extinguished from the infecting population, despite being beneficial. The death of a beneficial mutation can occur through a variety of mechanisms. For example, if a mutation is conditionally beneficial and offers a relative fitness advantage in only one type of environment within the host, and perhaps confers a severe fitness cost under other conditions, these conditions will hinder survival of the mutant. Alternatively, as observed in the LTEE, some beneficial mutations may simply be outcompeted by fitter clones due to clonal interference. Also, despite their relative advantage, clones that harbor beneficial mutations may still ultimately be eliminated by host immunity or antibiotic treatment. Finally, some mutations may confer a fitness advantage only in a tissue compartment within the host that does not permit transmission to a new host, thus creating an evolutionary “dead-end” [63,64]. Due to their relatively transient nature, these adapt-and-die mutations may escape detection, even though the information they encode is still valuable for understanding bacterial adaptation and mechanisms of pathogenesis. In this section, we argue that adapt-and-die mutations represent an untapped resource for information about bacterial evolution during human infection.

**Fig 3:**
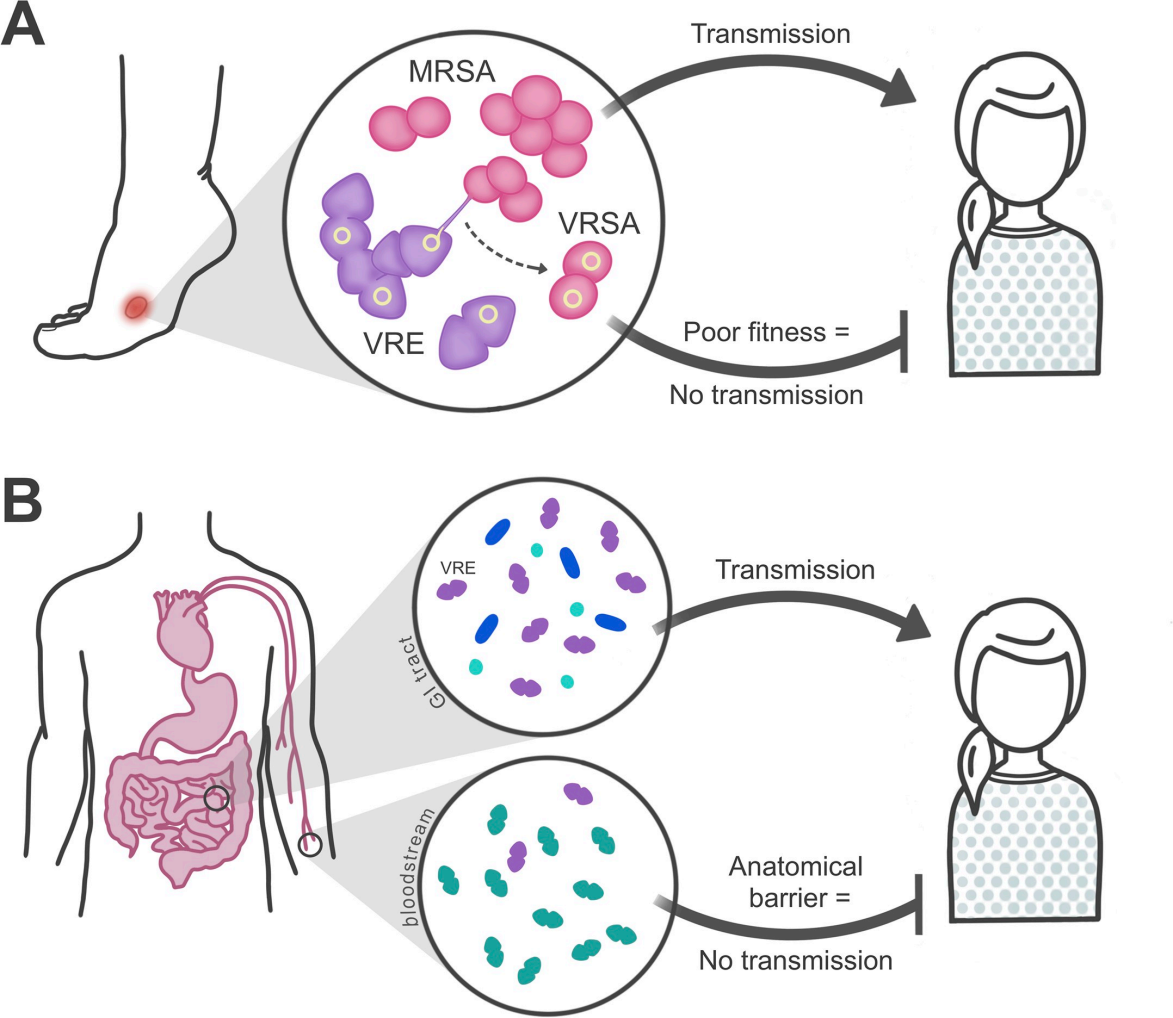
Bacterial evolution during acute infection. (A) Evolution of VRSA during coinfection with MRSA and VRE. While MRSA can be easily transmitted to other patients, VRSA have poor fitness and cannot be transmitted. (B) Different VRE populations in the GI tract and bloodstream of an infected patient. While VRE from the GI tract can be transmitted to other patients, VRE infecting the bloodstream are not transmitted. Conditionally beneficial mutations (shown in green) that are selected during growth in the bloodstream will similarly not be transmitted to other patients. Because anatomical barriers prevent conditionally beneficial adaptations from being transmitted, body sites such as the bloodstream can be considered evolutionary “dead-ends.” GI, gastrointestinal; MRSA, methicillin-resistant *S*. *aureus*; VRE, vancomycin-resistant *Enterococcus*; VRSA, vancomycin-resistant *S*. *aureus*.

The evolution of vancomycin-resistant *Staphylococcus aureus* (VRSA) is a notable example of an adapt-and-die scenario that generates a fitness cost restricting transmission to other hosts. *S*. *aureus* is a common colonizer of the CF airway, and several recent studies have compared *S*. *aureus* adaptation in CF patients to the adaptation of *P*. *aeruginosa* in the same environment [65–67]. *S*. *aureus* also frequently causes skin and soft tissue infections, bone and joint infections, and bacteremia. Vancomycin is the primary therapy for severe methicillin-resistant *S*. *aureus* (MRSA) infections, and its evolution to VRSA is concerning. Worldwide, there have been at least 52 VRSA isolates described since the first report in 2002 [68], yet epidemic spread has not occurred. VRSA is believed to evolve by conjugative transfer of an Inc18-like *vanA*-encoding plasmid from vancomycin-resistant enterococci (VRE) to MRSA. Detailed studies of a subset of cases have revealed that successful *vanA* plasmid transfer requires MRSA to harbor a pSK41-like conjugative plasmid [69]. The evolutionary jump from MRSA to VRSA can take place when VRE co-colonize the same anatomical site as a pSK41-positive precursor MRSA strain (Fig 3A). The clinical features of VRSA cases suggest that this most often occurs within a polymicrobial biofilm that is present, for example, on a skin wound or indwelling medical device [70]. Because the recipient MRSA strain has a low prevalence [71], *vanA* plasmid transfer between VRE and MRSA is a relatively rare event. Moreover, in the absence of vancomycin pressure, the *vanA* gene cluster causes a large growth defect in *S*. *aureus* [72]. Transmission of *S*. *aureus* from person to person occurs by direct contact and colonization of the skin. To date, no such VRSA transmission events have been reported, presumably due to the fitness cost imposed by the *vanA* operon (Fig 3A). Although rare, VRSA cases continue to be reported, thus future opportunities for the acquisition of compensatory mutations that might permit epidemic transmission remain a dangerous possibility.

A second notable example of the adapt-and-die scenario is when beneficial mutations are selected within tissue compartments that do not permit their transmission to new hosts (Fig 3B). Generally, bacterial transmission occurs via the respiratory route, fecal-to-oral route, or by direct skin contact. Although tissue compartments outside of a pathogen’s main transmission route, such as the bloodstream or central nervous system, are commonly invaded during infection, bacteria are generally unable to be transmitted directly from these compartments to other individuals. Furthermore, invasive infections are often caused by bacteria that colonize a different body site, such as the skin [73], nasopharynx [74], or gastrointestinal tract [75]. Due to the process of niche adaptation, these bacteria are often well adapted to reside at anatomical sites of colonization, rather than sites of infection. Because transmission does not happen between the bacterial populations at these more invasive sites of infection, beneficial traits must reevolve within each new patient. This process of evolution “on repeat” can be studied by sequencing pathogen genomes sampled from sites of colonization and infection within the same patient [76] or by sequencing the genomes of bacteria from outbreaks of a particular kind of infection [77,78]. One example of this is the enterococci, which are stable colonizers of the gastrointestinal tracts of land mammals [79]. In the antibiotic era, select enterococcal lineages have emerged that now cause invasive infections among immunocompromised and hospitalized patients [80]. One recent study of an outbreak of bacteremia caused by multidrug-resistant *Enterococcus faecalis* among 53 patients at a single hospital identified repeated adaptations in genes affecting cell surface polysaccharide production, including the enterococcal polysaccharide antigen (epa) and lipoteichoic acid [77]. In the latter case, independent mutations in the same transcriptional repressor were observed in 21 different patients. The mutated repressor was shown to cause overexpression of an enzyme that altered the abundance and structure of lipoteichoic acid; these changes conferred increased resistance to antibiotic treatment and innate immune stresses, which likely drove their occurrence [77]. Identical repressor mutations were observed in very few patients, strongly suggesting that these mutations were selected during bacterial growth in the human bloodstream and that they frequently died out instead of being transmitted to other patients (Fig 3B).

Similar to enterococci, *S*. *aureus* also adapt during the transition from skin colonization to invasive infection. Young and colleagues sequenced 1,163 *S*. *aureus* genomes from 105 patients with nose colonization that developed invasive infections of the bloodstream, soft tissues, or bones and joints [81]. Five different colonies were sampled per cultured site for each patient. This enhanced sampling was critical and enabled the discovery of over 1,000 de novo mutations with many examples of convergent evolution. Significant signatures of adaptation were observed in genes responding to the bacterial regulators *rsp* and *agr*, as well as genes that protect against host-derived antimicrobial peptides. *rsp* regulates the expression of surface antigens and toxins, while *agr* is involved in quorum sensing, toxin production, and abscess formation. Adaptive mutations associated with pathogenesis were more likely to occur in bacteria isolated from sites of infection compared to colonizing bacteria, and these mutations did not occur in *S*. *aureus* sampled from healthy individuals. Mutations associated with invasive infection of the bloodstream or joints in this study were likely of the adapt-and-die category, given that these tissue compartments are likely evolutionary “dead-ends.” In contrast, skin and soft tissue infections aid transmission, and there is evidence that adapt-and-live mutations arising at these sites can prime community outbreaks of *S*. *aureus* [78].

### Convergent evolution across species

Convergent evolution has been observed across different bacterial species, which demonstrates the generalizability of some in vivo adaptations. For example, mutations in *relA* that cause constitutive activation of the bacterial stringent response have been identified in persistent bloodstream infections caused by both *Enterococcus faecium* [82,83] and *S*. *aureus* [82–84]. Activation of the stringent response results in metabolic quiescence and antibiotic tolerance, thus promoting persistent infection [85]. Because strains carrying *relA* mutations all evolve in the bloodstream, exhibit growth defects, and show no evidence of transmission between patients, we would assign these mutations to the adapt-and-die category. Separately, a large analysis of published bacterial genomes recently uncovered several examples of common adaptive strategies found across different bacterial pathogens [54]. This dataset consisted of bacterial genomes from studies where the infecting pathogen was isolated and sequenced from at least 2 different time points from the same patient. It included over 7,000 isolates from 1,421 patients and encompassed 29 different bacterial species. Convergent adaptive changes across species often included common antigens recognized by the immune system, such as the flagellar filament, or those involved in antibiotic resistance and tolerance, such as mutations in RNA polymerase. By contrast, changes in virulence factors tended to be species specific. Overall, these findings suggest that the fate of an adaptive mutation is likely to depend on where and when it arises, as well as the species and type of infection in which it arises.

### Practical considerations for studying bacterial evolution in vivo in humans

As researchers, we are interested in studying how bacteria evolve in vivo during human infection in order to learn more about the basic biology of these organisms and to identify possible targets for therapeutic intervention. There are several limitations that currently hinder our ability to study in vivo bacterial evolution in sufficient depth to draw confident conclusions. In this section, we present what we consider to be practical considerations for studying bacterial evolution in vivo during human infection.

One major challenge to conducting rigorous studies of bacterial evolution in vivo is a lack of access to high-quality samples or the inability to collect the right sample at the right time. Studying bacterial evolution in vivo in humans requires the sampling of bacteria directly from human infections, which presents a formidable obstacle. While much can be learned from studying bacterial isolates collected during routine clinical care (which are usually considered discarded specimens and exempt from informed consent), such sample sets of convenience are likely to be sparse, incomplete, and/or biased in ways that limit their utility for conducting rigorous and well-controlled analyses. The choice of infection and sampling strategy should ideally be tailored to a set of research questions established at the outset of the study. Many researchers, however, are unable to consent and enroll patients in a clinical or interventional study, which leaves them to rely on the collection of whatever samples happen to be available for a particular organism or infection of interest. We propose that physicians and researchers should work together to collect samples from patients that (1) meet defined sets of inclusion criteria; (2) provide informed consent; and (3) can be sampled systematically and routinely to yield high-quality data for these studies.

A second major challenge that currently limits many studies of bacterial evolution in vivo is the reliance on sequencing and analysis of single clones of bacteria taken from an infection-derived clinical specimen. While the use of WGS to compare bacteria sampled from infected patients is a powerful approach for studying pathogen adaptation [3,86,87], the sampling of bacteria from infected patients nearly always involves the isolation of a single “representative” clone from the population of bacteria within the patient. This standard approach, while efficient and cost-effective, is based on the false assumption that infections are caused by clonal populations. The end result is a dramatic undersampling of bacterial genetic diversity within infected patients, which can lead to incorrect inferences about bacterial transmission and overlook low frequency variants that might be clinically relevant, such as antibiotic-resistant minority clones [87,88]. When bacterial clonal diversity within patients has been studied, only a small number of patients have been sampled [89,90]. This limits the conclusions that can be drawn from such studies and represents a critical barrier to progress in the field. Additionally, sampling the same patient longitudinally, even if over a short time period, adds another important dimension to these studies. We propose that sampling strategies should be tailored according to well-defined study questions. When it is possible and appropriate for the study, bacterial populations should also be collected longitudinally and should be sequenced alongside representative clones, as doing so is likely to yield additional insights beyond what can be learned from studying single bacterial isolates sampled from single time points.

A final challenge to conducting rigorous and well-controlled studies of bacterial evolution in vivo in humans is that appropriate tools for comparative and functional genomic analyses still need to be developed. A number of analysis tools have been developed for studying bacterial evolution in vitro and conducting genome-wide association studies in microbial pathogens [91,92], and many of these can be adapted to account for additional complexities that exist in human infections. One big hurdle that still remains is how to account for the movement of mobile genetic elements as well as recombination in these systems. In vitro experimental systems are largely genetically “closed,” meaning that new genetic material cannot enter the system and recombination is not a source of additional genetic variation in these settings (Fig 1). In vivo, however, we know that mobile element movement and recombination can play major roles in pathogen evolution [93,94], and these changes should ideally be analyzed alongside de novo mutations. Current approaches largely focus on genetic changes occurring within well-conserved genomic regions and are insufficient to detect genomic regions under selection that may not be highly conserved. Finally, comparative genomics approaches can only identify genes that appear to be under selection or mutations that appear to be beneficial for a particular organism. Functional studies are needed in order to test the hypotheses generated by comparative genomics analyses and to establish molecular mechanisms underlying beneficial adaptations. We propose that existing analysis tools should be adapted and new tools should be developed that can account for the added complexity of in vivo settings, for example, by incorporating pangenome analyses, using in vivo observations to develop computational models of infection, and refining these models to be able to predict the likely impact of observed mutations within a given infection context. The results of these analyses should then be considered as hypotheses that should be formally tested with functional follow-up approaches.

## Conclusions

We conclude this review with a few key points. First, microbes are constantly recording valuable information into their genomes in the form of mutations due to natural selection, and obtaining this information from clinical samples is now accessible to a growing body of researchers. Second, in vivo evolutionary dynamics during bacterial infections in humans are more complicated than in vitro experiments of microbial evolution, but uncovering signals of adaptation in this setting can result in important biological insights and uncover entirely new areas for investigation. Third, beneficial traits that arise during infection, but are short lived, are more challenging to identify, yet also provide critical insight into how bacteria adapt to new environments. And, finally, through this review, we hope to inspire clinicians and researchers alike to consider ways that they can move their research closer to the study of bacterial evolution in vivo in humans. Such studies certainly present numerous challenges compared to well-controlled in vitro evolution experiments. However, we believe that the potential benefits of in vivo studies of bacterial evolution during human infection are both highly impactful and directly translatable to improving the treatment and care of infected patients.
